# LEFTNet: low-frequency enhanced feature transmission network for dual-branch collaborative single image dehazing

**DOI:** 10.3389/frai.2026.1938382

**Published:** 2026-09-23

**Authors:** Beesetti Vanitha, Chintakindi Balaram Murthy

**Affiliations:** School of Computer Science and Engineering, VIT-AP University, Amaravati, Andhra Pradesh, India

**Keywords:** channel attention, cross-scale fusion, encoder–decoder, haze suppression block, image dehazing, image restoration, low-frequency representation

## Abstract

Image dehazing is a basic low-level vision task designed to recover high-fidelity images from hazed images. In autonomous driving, aerial surveillance, and outdoor scene recognition, image quality is significantly degraded by light attenuation due to scattering in the propagation medium, especially under hazy, foggy, or smoggy conditions. While remarkable advances have been made with existing deep learning methods, they remain constrained by over-smoothing, ineffective use of haze frequency, and insufficient consideration of multi-scale feature interactions. To meet these challenges, we introduce a novel single-image dehazing framework, called Low-Frequency Enhanced Feature Transmission Network (LEFTNet), which consists of four special modules: the Haze Suppression Block (HSB), the Dual-Branch Block (DBB), the Cross-Scale Fusion Block (CSFB), and the Channel Mining Mechanism (CMM). In the first stage, a hazy input image is decomposed into two complementary low-frequency representations: a haze-related component ($I_{\text{haze}}^{L}$) and a frequency-domain component ($I_{\text{freq}}^{L}$), which are jointly processed by the HSB for early, channel-selective haze attenuation. Then, the encoder extracts multi-scale hierarchical features, which are cleaned up at each stage by the Channel Mining module. The DBB's residual refinement enables intra-image global-to-local feature interactions, while the CSFB's learnable fusion coefficients enable inter-scale feature fusion. A dual fully connected bottleneck with Global Average Pooling (GAP) suppresses haze-dominant channels while enhancing structure-sensitive channels. Two symmetric branches ensure complementary representation learning, and a hybrid loss function comprising L1 loss and perceptual LSSIM loss assures both pixel-wise accuracy and perceptual fidelity. Comprehensive experiments on four benchmarks, RESIDE-6K, I-Haze, O-Haze, and NH-Haze, demonstrate that LEFTNet achieves competitive performance across the evaluated Peak Signal-to-Noise Ratio (PSNR), Structural Similarity Index Measure (SSIM), Average Mean Brightness Error (AMBE), Gradient Magnitude Similarity Deviation (GMSD), and Visual Saliency-Induced Index (VSI) metrics compared with recent dehazing methods.

## 1 Introduction

One of the most complex degradation issues in outdoor imaging systems is atmospheric haze. The degradation process begins with the scattering and absorption of light by suspended particles in the atmosphere, including smoke, aerosols, dust, mist, and water droplets. Light propagation in the atmosphere causes several scattering interactions that reduce a scene's radiance and produce airlight interference, leading to significant contrast loss, color distortion, edge smearing, and loss of scene structure. These degradations reduce visual quality and also significantly impact the ability of systems further down the pipeline to perform computer vision tasks, such as object detection, semantic segmentation, simultaneous localization and mapping (SLAM), autonomous navigation, intelligent surveillance, remote sensing interpretation, and robotic perception.

The physical process of forming haze has often been modeled by the Atmospheric Scattering Model (ASM), first defined by McCartney (1976) and later adapted for computer vision tasks by Narasimhan and Nayar (2000). Based on this model, the hazy image at pixel location x is modeled as


$$\begin{array}{l} I\left(x\right)=J\left(x\right)t\left(x\right)+A\left(1-t\left(x\right)\right) \end{array}$$ (1)

In Equation 1, *I*(*x*) is the observed hazy image, *J*(*x*) is the haze-free image, *t*(*x*) is the transmission map, and *A* is the global atmospheric light. This formulation offers a physical interpretation of the degradation of haze, but an accurate estimation of the atmospheric light and the transmission process is still very ill-posed because of the scene-dependent depth variation, inconsistent illumination, and heterogeneous haze distributions.

Early dehazing methods were mostly based on handcrafted priors and statistical assumptions. Kaiming et al. (2011) proposed the Dark Channel Prior (DCP), which is based on the observation that, under typical conditions, outdoor images without haze often have one or more low-intensity color channels in local regions. Later, several prior-model-based methods were proposed, including Color Attenuation Prior (CAP) (Qingsong et al., 2015), non-local haze-line modeling (Berman et al., 2016), contrast maximization (Tan, 2008), and probabilistic estimation of transmission (Fattal, 2008). However, they showed restoration potential, but their performance degrades under dense haze, sky dominance, night conditions, and a non-homogeneous atmosphere because the prior assumption is restrictive.

With the advent of deep learning, single-image dehazing studies have shifted to an end-to-end, data-driven approach, revolutionizing the field. Convolutional Neural Network (CNN)-based methods like DehazeNet (Cai et al., 2016), Multi-Scale CNN (MSCNN) (Ren et al., 2016), All-in-One Dehazing (AOD)-Net (Li et al., 2017), densely connected pyramid (DCPDN) (Zhang and Patel, 2018), Gated Fusion Network (GFN) (Ren et al., 2018), Multi-Scale Boosted Dehazing (MSBDN) (Dong et al., 2020), Two-Stage Multi-Domain (TSMD)-Net (Raju and Srinivas, 2024), and GridDehazeNet+ (Liu et al., 2023) gradually enhanced restoration results, utilizing residual learning, dense connectivity, multi-scale feature aggregation, and encoder–decoder optimization. Selectively highlighting informative spatial and channel responses using attention-guided approaches, such as the Feature Fusion Attention Network (FFA-Net) (Qin et al., 2020) or Detail-Enhanced Attention Network (DEA-Net) (Chen et al., 2024), further improved structural preservation. However, traditional CNN structures have inherent drawbacks, such as small receptive fields and limited ability to model long-range contextual relationships in large-scale haze distributions.

Recently, transformer-based dehazing networks have shown great success in capturing global contextual information through self-attention mechanisms. The vision transformer-based dehazing methods (Song et al., 2023; Yu et al., 2022) achieved improved perceptual restoration quality and enhanced long-range feature interactions compared with traditional CNN-based methods. However, conventional Transformer-based models establish attention relationships among a large number of spatial tokens, resulting in substantial computational and memory requirements as the image resolution increases. Such requirements may limit their practical deployment on resource-constrained platforms. In response to these challenges, recent dehazing research has increasingly explored frequency-aware representations and efficient global feature modeling. Motivated by the need for efficient feature interactions, the Low-Frequency Enhanced Feature Transmission Network's (LEFTNet's) cross-branch correspondence mechanism performs spatial matching along a single spatial dimension rather than constructing correspondences across the entire two-dimensional feature map. Although this formulation reduces the dimensionality of the correspondence computation, it still contains a quadratic component in the selected spatial dimension. The computational complexity and Floating Point Operations (FLOPs) of the proposed architecture are analyzed in detail to assess its computational requirements. Various frequency-guided dehazing methods have been proposed, such as frequency-domain dehazing and spatial dual-guidance for image dehazing (Yu et al., 2022), as well as adaptive frequency-guided dehazing (Hwang et al., 2024), which show that low-frequency components of images are heavily affected by haze. In contrast, the high-frequency image components contain important texture information. Explicit frequency decomposition has thus become a promising approach to extract information from the haze from the rest of the scene texture. Similarly, wavelet-guided restoration and spectral attention techniques (Hwang et al., 2024) are superior for preserving edges and reconstructing the perceptual image under dense haze conditions.

At the same time, lightweight transformer alternatives based on state-space modeling and linear attention have attracted significant interest. Long-range dependencies are efficiently modeled by Mamba-inspired architectures (Zhou et al., 2024) and linear attention dehazing networks (Qiu et al., 2023), which have substantially reduced computational complexity compared to standard self-attention networks. Additionally, hybrid CNN-transformer restoration methods (Zhao et al., 2021; Guo et al., 2022) leverage both local spatial representation learning and global contextual reasoning to improve dehazing. Recent diffusion-guided restoration models (Yu et al., 2023) have also shown strong capability in recovering realistic textures and perceptual details under extreme real-world haze conditions. Furthermore, lightweight, edge-oriented dehazing networks (Nuthi and Kankanala, 2025; Ullah et al., 2021) and multi-domain alignment strategies (Wang et al., 2024; Shao et al., 2020) focus on practical deployment and real-world generalization.

Despite these advances, several important limitations remain insufficiently addressed. First, most existing methods process haze entirely in the spatial domain without explicitly exploiting the spectral characteristics of atmospheric degradation. Second, standard encoder–decoder architectures propagate haze-contaminated shallow features through direct skip connections, reducing restoration purity. Third, haze-sensitive channel recalibration remains underexplored in current architectures. Fourth, transformer-based methods achieve strong global modeling capability at the expense of computational efficiency and scalability. Recent studies have also explored frequency-aware and feature interaction strategies for image restoration. Zhao et al. (2025) proposed a low-light stereo image enhancement and denoising method that exploits low-frequency information and cross-view feature interaction to improve detail recovery and noise suppression. However, their method relies on stereo image pairs, whereas single-image dehazing requires effective feature interactions from a single degraded input image.

The principal contributions of this work are summarized as follows:

- Low-frequency decomposition: This proposed framework explicitly decomposes the low-frequency information in the image from the structural-frequency information, enabling frequency-aware haze suppression.
- Haze suppression block (HSB): An easy-to-implement 1 × 1 channel recalibration technique that selectively blocks out the channels sensitive to haze and retains the texture-aware channels.
- Cross-branch interaction: The proposed Dual-Branch Block (DBB) allows efficient propagation of global-to-local features through using a residual mechanism for interactions at an acceptable level of computation as compared to full self-attention.
- Adaptive cross-scale fusion: The CSFB and CMM work together to enhance structural preservation and eliminate haze effects by learnable multi-scale feature fusion and hierarchical channel refinement.
- To achieve both perceptual fidelity and pixel-level reconstruction accuracy, a restoration system is optimized based on hybrid $L_{1}+L_{SSIM}$ supervision, and a symmetric dual-branch restoration architecture is proposed.

The outline of this article is organized as follows. Section 2 briefly reviews the related work on image dehazing. Section 3 presents the proposed LEFTNet architecture and its methodology in detail. Section 4 describes the experimental setup, datasets, evaluation metrics, and provides qualitative and quantitative analysis along with ablation studies. Finally, Section 5 concludes the article and discusses future research directions.

## 2 Related work

### 2.1 Physics-based and prior-driven dehazing methods

Existing image dehazing techniques mainly relied on a physical scattering model and handmade priors. McCartney (1976) laid the theoretical basis of atmospheric scattering for computer vision applications and Narasimhan and Nayar (2000). Kaiming et al. (2011) introduced the Dark Channel Prior (DCP) method for estimating the transmission maps in low-light haze images. Problems of estimating transmission and removing haze have been addressed in the study by Fattal (2008) with probabilistic independent component analysis and by Tan (2008) by solving a local contrast maximization problem. Qingsong et al. (2015) presented Color Attenuation Prior (CAP) and created a correlation between the brightness-saturation properties of the scene and its depth. Later, Berman et al. (2016) proposed non-local modeling of haze-lines using the RGB color clustering approach.

While prior-based methods have good physical interpretability and low training requirements, they are extremely sensitive to the restrictive assumptions of atmospheric homogeneity, image statistics, and illumination consistency. These techniques, therefore, tend to produce the halo effect, over-enhancement of colors, under-estimation of transmittance, and dramatic performance loss in dense haze and sky-dominant scenes.

### 2.2 CNN-based dehazing networks

The deep CNN-based methods have made substantial progress in image dehazing by learning the mapping from haze degradation end-to-end using extensive training data. DehazeNet (Cai et al., 2016) introduced deep transmission estimation using maxout feature extraction units. MSCNN (Ren et al., 2016) proposed a multi-scale coarse-to-fine haze removal network, and AOD-Net (Li et al., 2017) reformulated the haze removal model into an all-in-one parameter estimation network.

Later architectures began to emphasize dense connections, residual networks, and feature fusion. Densely connected pyramidal structures were used to estimate atmospheric light and transmission maps for the DCPDN jointly (Zhang and Patel, 2018). The Gated Fusion Network (GFN) (Ren et al., 2018) fused multiple derived inputs by confidence-aware fusion. MSBDN (Dong et al., 2020) improved the restoration quality by introducing dense feature fusion and a multi-scale boosting technique.GridDehazeNet+ (Liu et al., 2023) used multi-scale feature interaction via a grid to enhance the contextual representation.

The recent CNN-based approaches have investigated spectral decomposition, adaptive receptive fields, and lightweight residual attention. Another approach to haze removal is to leverage the spectral properties of haze, such as the separation of low-frequency atmospheric haze from high-frequency scene structure, which is explicitly performed in frequency-guided restoration networks (Yu et al., 2022). In addition, wavelet-based dehazing architectures (Hwang et al., 2024; Nuthi and Kankanala, 2025) have shown better edge preservation and texture recovery by learning frequency-domain representations. But the limited modeling capability for long-range dependencies remains in conventional CNNs with limited receptive fields.

### 2.3 Attention-based and transformer-based dehazing

Attention-guided feature recalibration has been an important paradigm in current image dehazing research. FFA-Net (Qin et al., 2020) proposed channel and pixel attention mechanisms to enhance the representations of the haze-sensitive regions selectively. The detail-enhanced attention modules are introduced to improve the representation of high-frequency features and to preserve structure in DEA-Net (Chen et al., 2024).

As a result, powerful alternatives to the transformer architecture became available for the modeling of global contextual information, namely transformer-based architectures. Self-attention mechanisms are effective in capturing long-range haze dependencies and large-scale illumination variations, as demonstrated by the DehazeFormer (Song et al., 2023) and related vision transformer-based dehazing networks (Zhao et al., 2021; Guo et al., 2022). To further enhance restoration capability, the hybrid CNN-transformer frameworks (Zhao et al., 2021) incorporated local CNN-based representation learning and global contextual reasoning.

Recently, lightweight transformer alternatives that approximate efficient attention and model the state space have become more popular. Linear attention dehazing networks (Qiu et al., 2023) aim to achieve computational efficiency while maintaining long-range contextual interactions. Similarly, Mamba-inspired state-space architectures (Zhou et al., 2024) offer an efficient sequence modeling capability, while requiring substantially less memory than traditional transformers. However, traditional transformer models often require extensive training data and computational resources, making them impractical for real-time, high-resolution applications.

### 2.4 Frequency-aware and generative dehazing methods

Recently, the study of frequency-domain restoration has become a very promising research field of image dehazing. Several recent works have made a conscious effort to perform spectral decomposition to separate information prevalent in the low-frequency components of images, which are dominated by haze. The Fourier-domain learning, frequency modulation, and frequency attention mechanisms are combined in spectral-aware dehazing frameworks, as discussed in the studies presented in Nuthi and Kankanala (2025) and Ullah et al. (2021), to enhance structural preservation and contrast restoration.

Methods of generative restoration have also shown good performance in recovering perceptually realistic details. Diffusion dehazing approaches (Yu et al., 2023) are based on iterative denoising priors. Diffusion dehazing (Yu et al., 2023) is based on iterative denoising priors, which gradually produce haze-free images, capturing more natural color consistency and texture detail. Multi-domain feature alignment techniques (Wang et al., 2024; Shao et al., 2020) address the domain discrepancy between synthetic and real-world haze patterns through domain-adaptive feature regularization and domain-invariant representation learning.

Although many of the generative and spectral-domain methods are effective, they are often computationally expensive and difficult to optimize for lightweight deployment scenarios.

### 2.5 Positioning of LEFTNet

The existing prior-based, CNN-based, transformer-based, and frequency-aware dehazing methods are fundamentally different from LEFTNet. Unlike the previous manual and restrictive atmospheric methods, LEFTNet makes no assumptions about the atmosphere, nor does it require manual design for statistical observation. In contrast to traditional CNN architectures, LEFTNet explicitly models haze degradation in the frequency domain as low-frequency decomposition. The proposed Dual-Branch Block (DBB) enables efficient global-to-local interactions at substantially lower computational cost than full self-attention, as demonstrated by the complexity analysis in Table 1.

**Table 1:** Computational cost comparison of DBB and full self-attention.

Scale (*H, W, C*)	DBB (GMACs)	Full self-attention (GMACs)	Reduction
(512, 512, 16)	6.44	2,199.02	341 ×
(256, 256, 32)	1.61	274.88	171 ×
(128, 128, 64)	0.40	34.36	85 ×
Total	**8.46**	**2,508.26**	**297 × **

In addition, the LEFTNet adopts a symmetric dual-branch structure comprising four complementary modules: frequency-aware decomposition, haze-sensitive channel suppression, adaptive cross-scale fusion, and hierarchical channel mining. This common design helps achieve better perceptual quality, structural consistency, and computational efficiency under both simulated and actual atmospheric conditions. Recent transformer- and frequency-based dehazing architectures have further advanced benchmark performance. Song et al. (2023) (dehazing transformer [DehazeFormer]) and Qiu et al. (2023) (Multi-Branch Transformer expanded by Taylor formula [MB-TaylorFormer]) employ vision-transformer backbones to achieve strong Peak Signal-to-Noise Ratio (PSNR)/Structural Similarity Index Measure (SSIM) on synthetic benchmarks, while lightweight alternatives such as WaveDH (Hwang et al., 2024) and RSDehamba (Zhou et al., 2024) target efficient, wavelet- or Mamba-based dehazing. Most recently, TSMD-Net (Raju and Srinivas, 2024) and dual-path spatial-frequency fusion networks (Wang et al., 2024) have explored two-stage and frequency-aware designs conceptually related to our low-frequency decomposition strategy. These transformer-based methods typically require substantially larger parameter budgets than convolution-based designs. They are predominantly evaluated on a relatively homogeneous synthetic haze (the Synthetic Objective Testing Set [SOTS]). In contrast, LEFTNet targets a lightweight (2.75 M parameters), dual-branch design explicitly suited to non-homogeneous and real-world haze conditions (NH-HAZE, RESIDE-6K, I-Haze, and O-Haze).

## 3 Proposed method

### 3.1 Overall framework

LEFTNet is a single-image dehazing network based on an encoder-decoder structure with four new modules, as depicted in Figure 1: HSB, DBB, CSFB, and channel mining (CM). A haze suppression algorithm, called the Haze-Sensitive Band (HSB), is used to suppress haze-dominant channels after extracting low-frequency representations from the input *I*. These features pass through a set of encoder blocks comprising integrated Channel Mining modules, and are then enhanced by the DBB for cross-branch interaction and by the CSFB for adaptive multi-scale fusion. The decoder reconstructs a dehazed image based on the enhanced representation. The mirrored right branch receives The mirrored right branch receives the same hazy input; the Encoder branches share weights (θ_*left*_ = θ_*right*_) across both branches, and beyond the Encoder the two branches interact only through the DBB, which establishes cross-branch correspondence between *S*_*left*_ and *S*_*right*_ before the CM module refines them into *M*_*left*_ and *M*_*right*_. Formally, the input *I* is decomposed into:


$$\begin{array}{l} I=I_{L}^{haze}+I_{L}^{freq} \end{array}$$ (2)

where $I_{L}^{haze}$ represents the haze-related low-frequency component, while $I_{L}^{freq}$ denotes the frequency-domain component containing structural details and texture information. To operationalize this decomposition, $I_{L}^{haze}$ is directly estimated from the input image using a learned convolutional encoder *E*_θ_, while $I_{L}^{freq}$ is derived via residual subtraction:


$$\begin{array}{l} I_{L}^{haze}=\sigma \left({\mathrm{Conv}}_{1\times 1}\left(E_{\theta }\left(I\right)\right)\right) \end{array}$$ (3)

$$\begin{array}{l} I_{L}^{freq}=I-I_{L}^{haze} \end{array}$$ (4)

where σ denotes the Sigmoid activation function, ensuring normalized intensity values within [0, 1].

**Figure 1:**
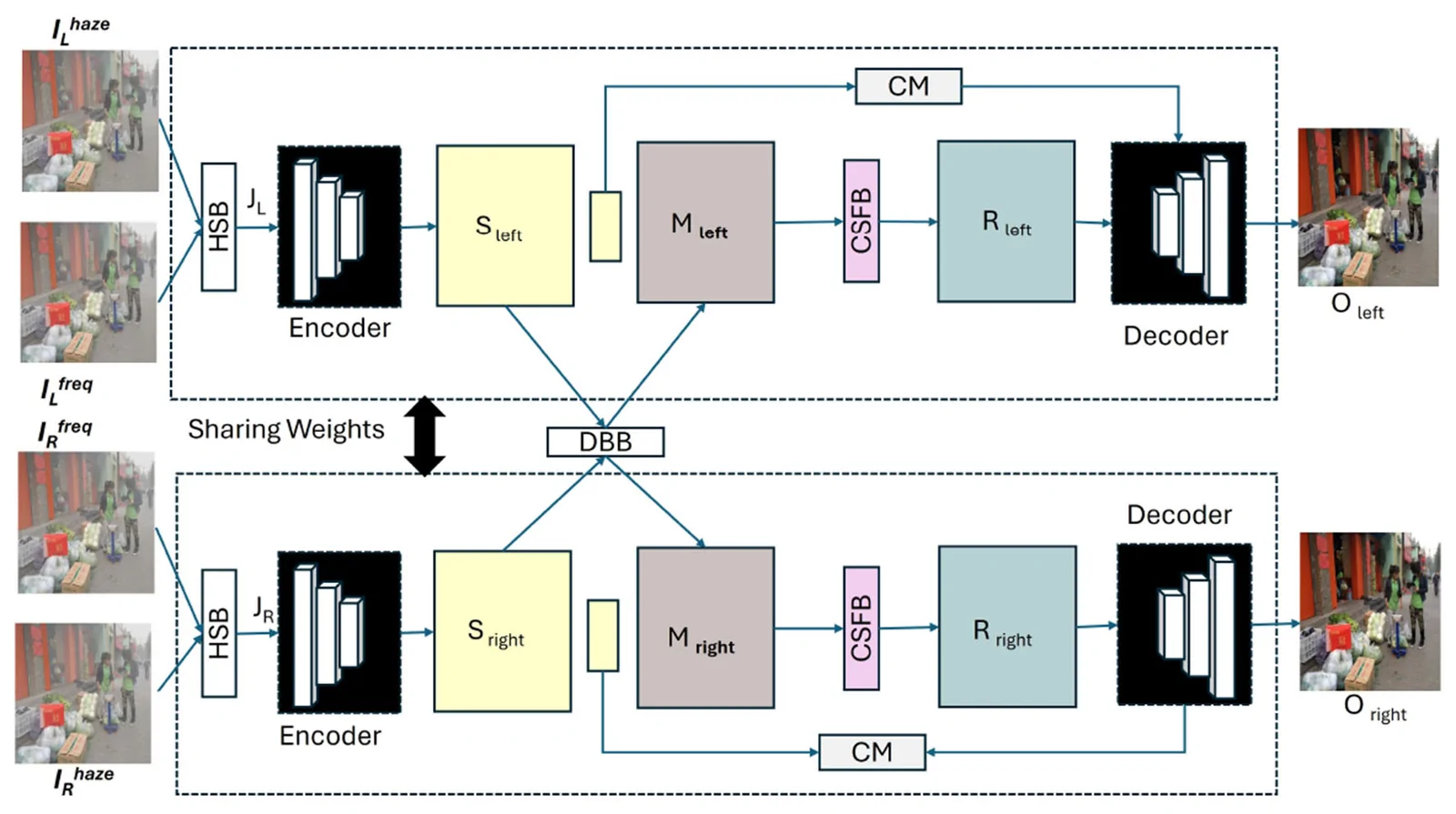
Overall architecture of the proposed LEFTNet for dual-branch collaborative single-image dehazing.

### 3.2 Low-frequency representation and haze suppression block

Atmospheric haze mainly affects low spatial frequencies in images. Diffuse scattering affects global illumination and color balance more than high-frequency information such as edges and fine textures. The haze veil is itself an additive low-frequency component.

The HSB processes these isolated components and computes its output feature map *F*_*HSB*_ as:


$$\begin{array}{l} F_{HSB}=\delta \left(W_{1}*I_{L}^{haze}+W_{2}*I_{L}^{freq}\right) \end{array}$$ (5)

where *W*_1_ and *W*_2_ represent learnable convolution kernels, * denotes the convolution operation, and δ represents the non-linear activation function. The two low-frequency inputs are then combined and passed through a 1 × 1 convolution that reorganizes and compresses the channel information, as illustrated in Figure 2. Then, Global Average Pooling (GAP) generates a scalar descriptor for every channel *c*:


$$\begin{array}{l} g_{c}=\frac{1}{H\times W}\overset{H}{\underset{i=1}{\sum }}\overset{W}{\underset{j=1}{\sum }}F\left(x,y\right) \end{array}$$ (6)

where *H* and *W* are the spatial dimensions, and *F*_*c*_(*x, y*) is the feature value at location (*x, y*) in channel *c*. The descriptor vector *g* is fed through a Rectified Linear Unit (ReLU) activation and a 1 × 1 convolution to obtain channel importance weights:


$$\begin{array}{l} F_{\text{weighted}}=w_{c}⊙F_{c} \end{array}$$ (7)

**Figure 2:**
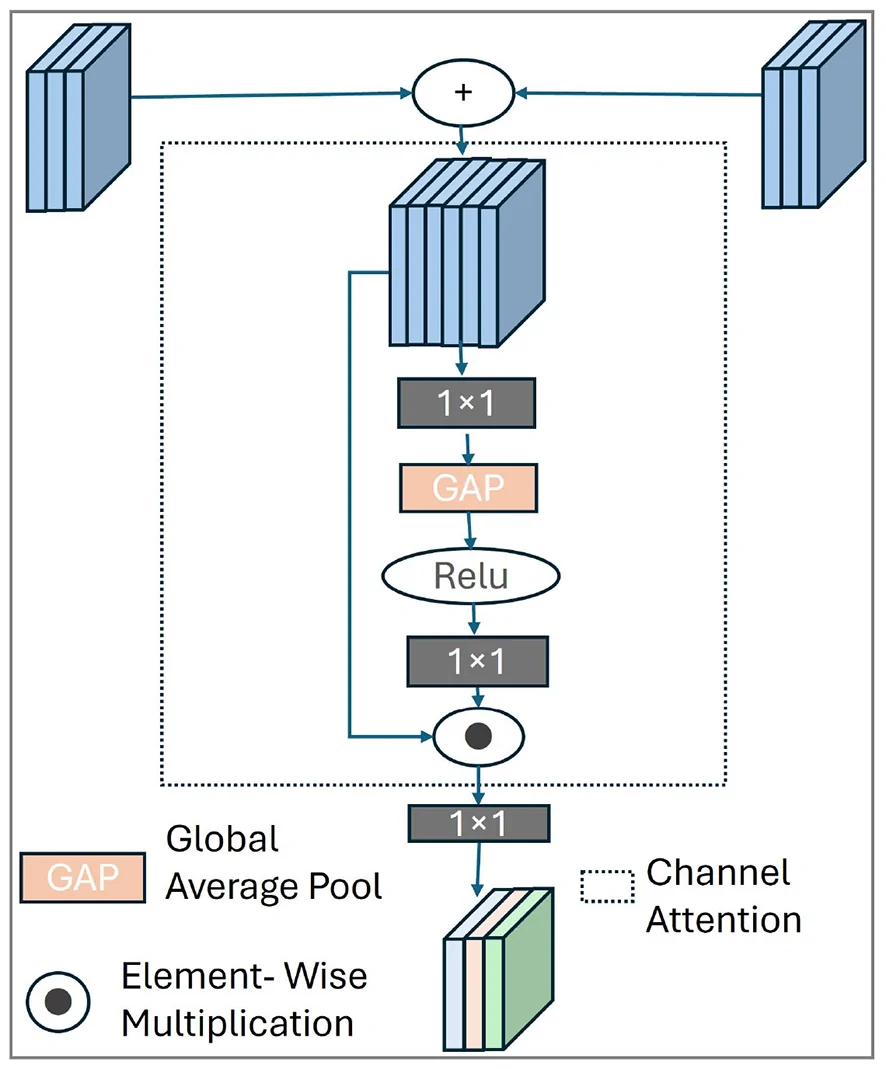
Detailed architecture of the Channel Mining (CM) module.

The learned importance weight per channel *c* is denoted as *w*_*c*_, and element-wise multiplication is denoted as ⊙. Low weights are assigned to haze-dominant channels, while the structure-sensitive channels are preserved.

### 3.3 Encoder design with channel mining integration

The proposed architecture of the encoder–decoder module and the design of the proposed encoder residual block are shown in Figures 3, 4, respectively. The hazy image shown on the right side of the figure is iteratively passed through a series of encoder modules that extract the features at different spatial scales. For the (*i*)-th stage, let *E*_*i*_(·) be the encoder function and ${\text{S}}_{i}^{\text{enc}}$ the feature map for the (*i*)-th stage. The number of feature channels is progressively upsampled in deep networks to capture more semantic information, while the spatial resolution is progressively downsampled. Shallow encoder layers primarily extract low-level visual information such as edges, texture, local contrast variations, and fine-grained structural details. The deeper layers of the encoder, however, acquire high-level contextual information such as the global haze distribution, the geometry of a scene, and the large-scale structural information.

**Figure 3:**
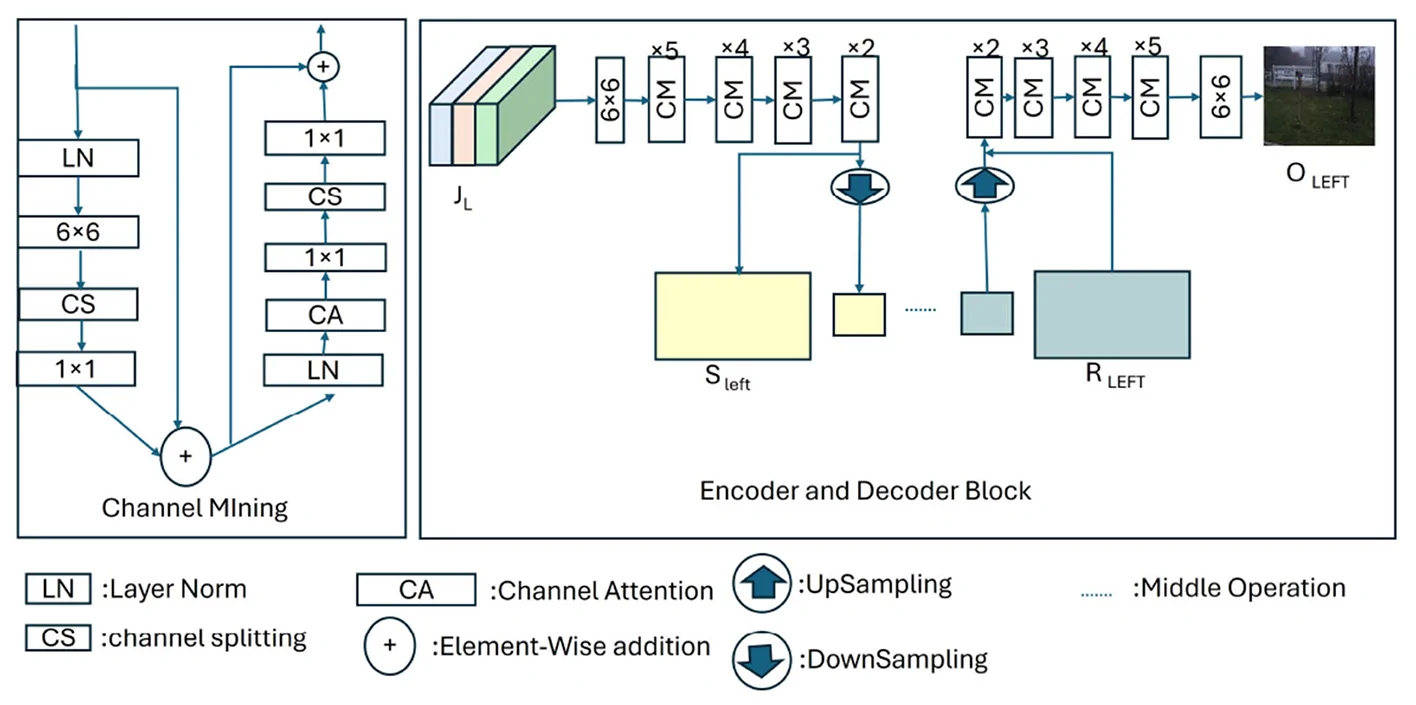
Detailed architecture of the Channel Mining (CM) module and its integration within the Encoder–Decoder Block.

**Figure 4:**
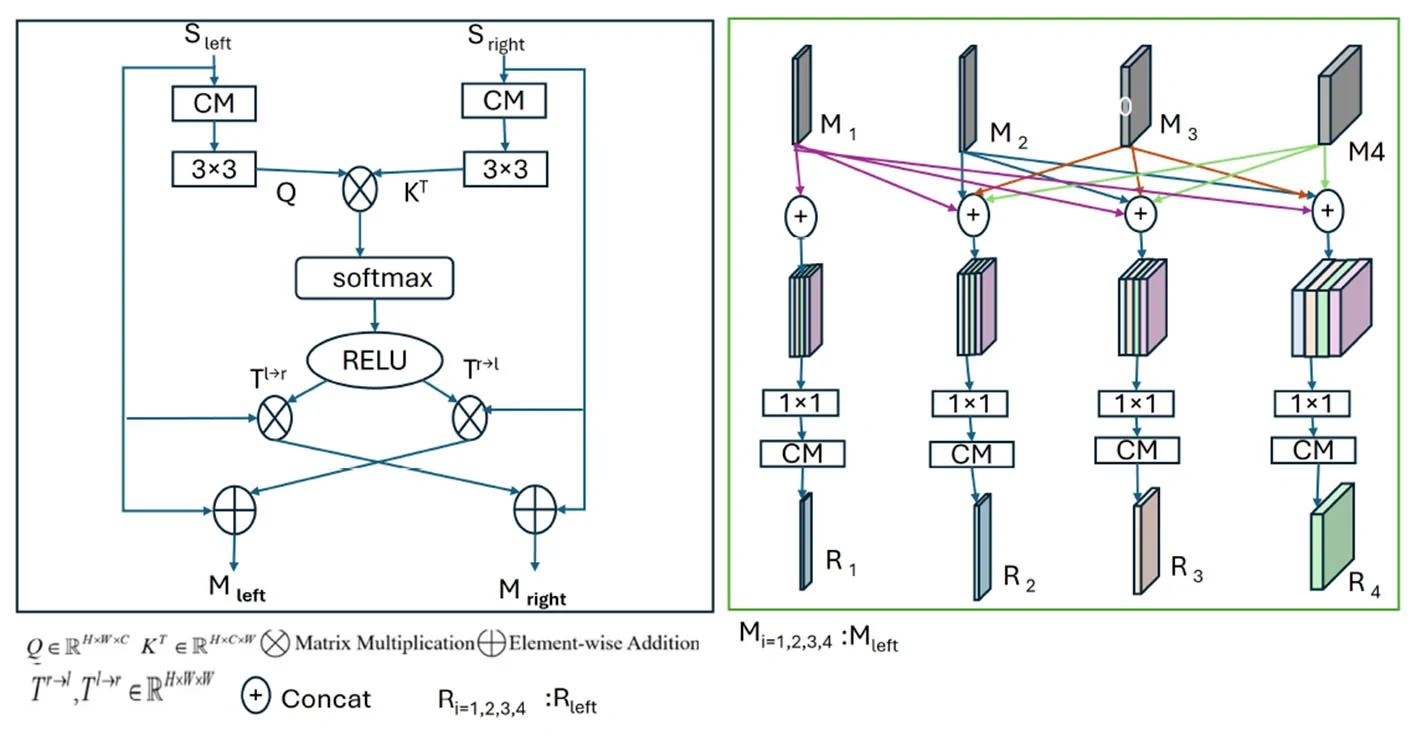
Detailed architecture of the Dual-Branch Block (DBB) and the Cross-Scale Fusion Block (CSFB).

A Channel Mining Module (CMM) is added to each encoder stage to discriminate features at each resolution level. The module selectively highlights informative answers and dampens unnecessary activation channels. As a result, the extracted features are more representative and are passed to the next stage to further enhance their quality and enable better haze removal.

Multi-scale encoder-generated feature maps are then transferred to the decoder via skip connections after going through the bottleneck layer. The decoder gradually reconstructs the spatial resolution by applying upsampling operations and incorporating the encoder's features of the same scale. This U-Net-like design keeps all the details of the images and can perform well in fusing low-level information and high-level information, and finally outputs a high-quality dehazed image with fine details preserved.

On the left, we show the architecture of a single encoder residual block. At the same time, the input feature map is fed into two parallel convolutional streams, each consisting of stacked convolutional layers, with 1 × 1 bottleneck projections in each layer for efficient feature transformation. The two branches are combined at the branch level with a residual addition. Moreover, another residual addition is added between the block input and output. This nested residual design helps achieve efficient feature reuse, better gradient propagation, reduced vanishing-gradient problems, and expanded the feature space of the encoder network.

The encoder extracts multi-scale hierarchical features from *F*_*HSB*_:


$$\begin{array}{l} S_{\text{left}}=E_{i}\left(F_{HSB}\right) \end{array}$$ (8)

Let *E*_*i*_(·) represent the encoder operation when scaled by *i*. With depth, spatial resolution decreases, and the number of channels increases, resulting in increasingly abstract haze representations. The Channel Mining module is after each encoder stage and has two functions: (i) refine channel responses at the current stage and (ii) provide calibrated features to the next stage. To guarantee gradient stability and feature reuse, two parallel convolutional branches with 1 × 1 bottleneck projections are created in each encoder residual block, followed by nested residual additions at both the branch and block levels.

### 3.4 Dual-branch block

The DBB facilitates cross-branch interaction by communicating global haze features between the left and right branches at each spatial scale, refining local structures through learned inter-branch correspondence. Given left- and right-branch scale features $S_{left},S_{right}\in {\mathbb{R}}^{H\times W\times C}$, the query and key representations are computed as:


$$\begin{array}{l} Q=\text{Con}{\text{v}}_{3\times 3}(\text{CM}\left(S_{left}\right))\in {\mathbb{R}}^{H\times W\times C} \end{array}$$ (9)

$$\begin{array}{l} K=\text{Con}{\text{v}}_{3\times 3}(\text{CM}\left(S_{right}\right))\in {\mathbb{R}}^{H\times W\times C} \end{array}$$ (10)

where CM(·) denotes the Channel Mining Mechanism (Section 3.6). Cross-branch correspondence maps are obtained via:


$$\begin{array}{l} T^{l\to r}=\text{ReLU}(\text{Softmax}\left(QK^{T}\right))\in {\mathbb{R}}^{H\times W\times W} \end{array}$$ (11)

$$\begin{array}{l} T^{r\to l}=\text{ReLU}(\text{Softmax}\left(KQ^{T}\right))\in {\mathbb{R}}^{H\times W\times W} \end{array}$$ (12)

Each correspondence map is matrix-multiplied with the opposite branch's original scale feature and combined with a residual connection to produce the Matched Features:


$$\begin{array}{l} M_{left}=S_{left}+T^{l\to r}S_{right} \end{array}$$ (13)

$$\begin{array}{l} M_{right}=S_{right}+T^{r\to l}S_{left} \end{array}$$ (14)

The DBB is the only point of communication between branches, allowing controlled exchange of information without collapsing both branches to identical representations.

### 3.5 Cross-scale fusion block

In real haze environments, haze distributions are highly non-uniform and vary considerably across space and scene depth layers. Therefore, features extracted from different encoder scales provide complementary information about local textures, object boundaries, global scene structure, and haze concentration. Accurate estimation and restoration of haze are thus crucial, where integration of these multi-scale representations is essential.

The proposed Cross-Scale Fusion Block (CSFB) is designed to fuse features across scales adaptively. Define the set of feature maps to be refined by DBB as ${\left\{R_{\text{left}}^{i}\right\}}_{i=1}^{N}$, where *N* is the number of encoder stages.

The CSFB fuses these representations by means of learnable scale coefficients, given in Equation 12. Unlike fixed fusion strategies, the learnable coefficients α_*i*_ allow the network to adaptively assign the importance of each scale during the training process. The model can be fine-tuned to better highlight small-scale features in scenes where it is important to preserve structural detail, or to better highlight larger-scale features in scenes where it is important to estimate large-scale haze distributions and scene context.

The feature maps at different encoder stages have distinct spatial resolutions prior to fusion. Therefore, bilinear upsampling is used to match all feature representations to the same resolution, ensuring consistent scale for feature aggregation. Then the corresponding fusion coefficients are added to the aligned features, forming a fused multi-scale representation. A dynamically fusing mechanism is used for effective transmission of complementary information across various receptive fields with a lightweight computational design.

The general architecture of the CSFB is shown in Figure 4 (right) for the case of (*N* = 4) encoder scales. The four feature maps from the encoders are then fed into the fusion module. All-to-all feature interaction is implemented via dense cross-scale connections at each fusion node, which receives weighted contributions from all encoder scales. This high-quality aggregation enables each scale to access fine-grained local information and high-level contextual cues from other scales. This fused feature map thus shows greater contextual awareness and feature consistency at various spatial scales.

After scale fusion, a 1 × 1 convolution layer is applied to each aggregated feature map to do channel mixing and dimensional refinement. The enhanced informative feature channels and suppressed redundant responses are then passed to the Channel Mining Mechanism (CMM) for further enhancement of informative feature channels and suppression of redundant responses. Finally, the multi-scale representations are fed into the respective decoder stages via skip connections, which allow the decoder to produce more structurally faithful, contrast-preserving, and haze-removing dehazed images.

Spatial variations in location and different layers of scene depth exist in haze density. This is addressed by the CSFB through adaptive scale-weighted feature fusion:


$$\begin{array}{l} O_{\text{left}}=\overset{N}{\underset{i=1}{\sum }}\alpha_{i}\cdot R_{\text{left}}^{i} \end{array}$$ (15)

The end-to-end learnable fusion coefficient α_*i*_ applies to scale *i*, and *N* is the number of scales in the encoder. Spatially aligns all feature maps to the same resolution prior to fusion, using bilinear upsampling. The *N* = 4 encoder scales perform an all-to-all weighted summation with α_*i*_ followed by a 1 × 1 convolution and a Channel Mining block prior to the encoder scale-specific outputs being fed to the decoder.

### 3.6 Channel mining mechanism

Channel Mining Mechanism is a second-stage channel-wise recalibration after CSFB. It first accesses the global statistics through GAP:


$$\begin{array}{l} z_{c}=\frac{1}{H\times W}\overset{H}{\underset{x=1}{\sum }}\overset{W}{\underset{y=1}{\sum }}O_{\text{left}}^{c}\left(x,y\right) \end{array}$$ (16)

Encoding spatial information into a single parameter per channel. A dual fully-connected bottleneck calculates the weights for the channels:


$$\begin{array}{l} w=\sigma \left(W_{2}\cdot \delta \left(W_{1}\cdot z\right)\right) \end{array}$$ (17)

The learnable weight matrices *W*1 ∈ ℝ^(*C*/*r*) × *C*^, *W*2 ∈ ℝ^*C*×(*C*/*r*)^ are reduction ratio *r* = 16. ReLU is denoted as δ(·), and the sigmoid function is denoted as σ(·). The bottleneck preserves inter-channel dependencies and reduces the number of parameters; the sigmoid maps weights to the range [0,1] for soft channel gating. The refined output feature map is obtained from a residual formulation:


$$\begin{array}{l} {\bar{O}}_{\text{left}}=O_{\text{left}}+w⊙O_{\text{left}} \end{array}$$ (18)

The channel-weighted component represents learned adjustments, and the residual addition preserves the original feature information.

### 3.7 Decoder block and image reconstruction

The decoder gradually upsamples the refined feature representation to restore the full spatial resolution. There is a convolution for refinement associated with each upsampling block to compensate for artifacts introduced by upsampling. The dehazed output is achieved as:


$$\begin{array}{l} J_{L}=D\left({Ō}_{\text{left}}\right) \end{array}$$ (19)

The decoder pipeline consists of upsampling, feature aggregation, a final 1 × 1 convolution followed by a sigmoid activation to ensure pixel values remain within the range [0,1] [where *D*(·) denotes the decoder pipeline]. Channel Mining skip connections connect the encoder's CM modules to the decoder's stages, providing coarse structural detail to recover fine detail.

### 3.8 Symmetric right branch

A symmetric right branch (SRB) shares learnable parameters with the left branch. It operates on the same hazy input to generate a parallel dehazed output *J*_*R*_, corresponding in role to the left-branch output *J*_*L*_, as defined below. The correspondence of the notation is:


$$\begin{array}{l} I_{L}↔I_{R},\;O_{\text{left}}↔O_{\text{right}},\;J_{L}↔J_{R} \end{array}$$ (20)

The modules (HSB, Encoder, DBB, CSFB, CMM, and Decoder) are shared between both branches. This symmetry allows both branches to apply the same learned transformation to the hazy input while maintaining separate intermediate feature representations. The DBB is the sole point of communication between the branches, enabling bidirectional interactions between their corresponding encoded representations. Specifically, the DBB establishes learned inter-branch correspondence at each spatial scale and transfers complementary feature information from one branch to the other through residual feature refinement.

Thus, the two branches do not simply generate independent outputs for implicit ensembling; instead, their intermediate representations are collaboratively refined through the DBB while preserving the shared-weight structure. The effectiveness of this dual-branch design is further evaluated through the single-branch and dual-branch ablation reported in Table 2.

**Table 2:** Computational complexity comparison of single-branch and dual-branch models.

Model	Params (M)	MACs (G)	FLOPs (G)	Peak GPU memory (GB)	Runtime (ms)
Single-branch (no DBB)	2.486	91.35	182.69	0.41	163.59
Dual-branch (full model, with DBB)	2.747	210.80	421.60	1.70	409.98

### 3.9 Loss function

LEFTNet employs a hybrid loss function combining $L_{1}$ loss and $L_{SSIM}$ loss:


$$\begin{array}{l} L_{H}=L_{1}+L_{SSIM} \end{array}$$ (21)

$L_{1}$ Loss. The $L_{1}$ (Mean Absolute Error) loss measures the average absolute error between the recovered image from the hazed image and the clear image *Y* at the pixel level:


$$\begin{array}{l} L_{1}=\frac{1}{N}\overset{N}{\underset{i=1}{\sum }}|{ŷ}_{i}-y_{i}| \end{array}$$ (22)

$L_{SSIM}$ Loss. These are pixel-wise losses that minimize the absolute reconstruction error but do not directly optimize for perceptual quality or structural similarity. The Structural Similarity Index Measure (SSIM) (Zhou et al., 2004) compares images as a whole based on three factors: luminance *l*, contrast *c*, and structure *s*.


$$\begin{array}{l} SSIM\left(x,y\right)={\left[l\left(x,y\right)\right]}^{\alpha }\cdot {\left[c\left(x,y\right)\right]}^{\beta }\cdot {\left[s\left(x,y\right)\right]}^{\gamma } \end{array}$$ (23)

Following the standard SSIM definition, the weighting parameters in Equation 23 are set to α = 1, β = 1, and γ = 1. These components represent the local means, variances, and covariance, yielding an SSIM value bounded between -1 and 1 (where -1 indicates no similarity and 1 denotes perfect structural similarity). Accordingly, the SSIM loss is defined as:


$$\begin{array}{l} L_{\text{SSIM}}=1-\text{SSIM}\left(ŷ,y\right) \end{array}$$ (24)

The hybrid approach combines the advantages of both loss functions: $L_{1}$ enforces the reconstruction error to be as close as possible to the original signal at each pixel, while $L_{SSIM}$ ensures that the reconstructed signal has perceptual characteristics and structural similarity with the original signal. They work together to create outputs that are both mathematically correct and aesthetically pleasing.

## 4 Results and discussion

### 4.1 Experimental setup

#### 4.1.1 Datasets

The synthetic hazy images generated by the atmospheric scattering model with different parameters applied to clean background images from the RESIDE benchmark (Li et al., 2019) are realistic. The Indoor Training Set (ITS) and Outdoor Training Set (OTS) are concatenated and randomly sub-sampled to obtain a RESIDE-6K split of 6,000 hazy/clean image pairs, with 5,000 pairs for training and 1,000 pairs for testing. To compare fairly with state-of-the-art techniques, two SOTS subsets are used: Indoor and Outdoor.

I-Haze (Ancuti C. et al., 2018) is an indoor hazy image dataset of controlled hazy images, created from clean reference images. It challenges approaches for recovering details of the indoor scene, such as fine textures and object structures, that are partially hidden by haze.

O-Haze (Ancuti C. O. et al., 2018) is a set of outdoor images with optically thick haze, one of the most difficult sets of images to dehaze. The NH-Haze (Ancuti et al., 2020) is a collection of outdoor images taken with a camera under non-homogeneous (spatially varying) haze conditions. It is an important benchmark for assessing robustness to real-world haze variability, as it includes 55 image pairs with clean reference images obtained through a dedicated, calibrated acquisition pipeline.

#### 4.1.2 Implementation details

All experiments were carried out in Python 3.10, PyTorch 2.0.1, and CUDA 11.8 on a workstation with an NVIDIA graphics processing unit (GPU). The images were resized and cropped to the central 512 × 512 pixels. The training used random 128 × 128 patch crops from full-resolution pairs at a batch size of 8, which produced a good balance between gradient noise and gradient accuracy. Adam was used with its hyperparameters $\beta_{1}=0.9,\beta_{2}=0.999,\epsilon =1\times 10^{-8}$ and a cosine annealing learning rate of 1 × 10^−4^ for 300 epochs. To enhance generalization, we adopted data augmentation techniques, including random horizontal and vertical flips, each with probability 0.5.

#### 4.1.3 Evaluation metrics

Perceptual and structural quality metrics for dehazed images differ, and no single criterion can fully capture the various aspects of the dehazing algorithm's performance. Hence, a range of complementary objective metrics is used to offer a well-rounded and accurate evaluation of the proposed dehazing paradigm. The fidelity of reconstruction is evaluated by calculating the Peak Signal-to-Noise Ratio (PSNR) (Hore and Ziou, 2010) in decibels (dB), which quantifies the pixel-wise similarity between the restored image and the ground-truth image; higher PSNR values indicate lower reconstruction error and thereby better image quality. Structural Similarity Index Metric (SSIM) (Zhou et al., 2004) measures the ability to preserve structural information by comparing the luminance, contrast, and structural consistency of the restored and reference images, and the closer the value to 1, the better the structural similarity. Gradient Magnitude Similarity Deviation (GMSD) (Xue et al., 2014) quantifies the uniformity of edge and texture preservation by calculating the difference between the gradient magnitudes of the recovered image and the reference image, and lower GMSD values indicate higher uniformity, which means better visual consistency and edge preservation. Average Mean Brightness Error (AMBE) (Soong-Der, 2003) quantifies the deviation in average luminance between the enhanced image and its reference values, with smaller values indicating more accurate brightness retention. Visual Saliency-Induced Index (VSI) (Zhang et al., 2014) assesses image quality by leveraging saliency-aware features that align with human visual perception, where larger values correspond to better perceptual similarity.

### 4.2 Quantitative analysis

We quantitatively compare LEFTNet with 11 state-of-the-art methods: All-in-One Dehazing Network (AOD-Net), Artificial Multi-Exposure Image Fusion Network (AMEI-Net), Artificial Multi-Exposure Image Fusion (AMIF Net), Region-Adaptive Defogging and Enhancement (RADE), Contrast Enhancement and Exposure Fusion (CEEF), MSCNN, Aerial Image Dehazing with Attentive Deformable Transformers (AID-Net), Detail-Enhanced Convolution and Content-Guided Attention (DEA-Net), Priors-Assisted Dehazing Network (PAD-Net), Selective Wavelet Attentive M-Network+ (SWAM Net+), and Full-Spectral Self-Attention Detail Enhancement Network (SAD-Net).

RESIDE-6K. Among all methods, the highest PSNR, 28.76 dB, is achieved by LEFTNet, as shown in Table 3. It also achieves the best AMBE score of 4.80, indicating better brightness fidelity. However, LEFTNet does not achieve the best SSIM or GMSD on this dataset: PAD-Net attains a higher SSIM (0.903 vs. 0.824) and a lower (better) GMSD (0.044 vs. 0.082), suggesting stronger structure-preservation on this particular benchmark. These results indicate that while LEFTNet excels at haze removal and brightness restoration, its structural-fidelity performance on RESIDE-6K leaves room for improvement, which we discuss further in Section 4.6.

**Table 3:** Quantitative comparison on the RESIDE-6K dataset.

Method	PSNR↑	SSIM↑	AMBE↓	GMSD↓	VSI↑
AOD-Net (Li et al., 2017)	15.72	0.771	31.30	0.092	0.965
AMEI-Net (Zhu et al., 2021)	18.65	0.776	22.25	0.074	0.986
AMIF-Net (Galdran, 2018)	18.72	0.822	16.32	0.064	0.977
RADE (Li et al., 2021)	16.10	0.715	19.86	0.146	0.939
CEEF (Liu et al., 2022)	17.62	0.730	22.28	0.089	0.967
MSCNN (Ren et al., 2016)	18.45	0.824	18.07	0.057	0.979
AID-Net (Kulkarni and Murala, 2023)	12.69	0.621	45.92	0.158	0.921
DEA-Net (Chen et al., 2024)	16.75	0.795	20.41	0.076	0.976
PAD-Net (Yi et al., 2024)	22.93	**0.903**	11.56	**0.044**	**0.989**
SWAM-Net+ (Nuthi and Kankanala, 2025)	25.69	0.821	6.21	0.065	0.982
SAD-Net (Niu et al., 2025)	26.43	0.832	7.29	0.072	0.972
LEFTNet (Ours)	**28.76**	0.824	**4.80**	0.082	0.979

Best results in bold.

I-Haze Table 4. Our proposed method achieved PSNR and SSIM of 22.28 dB and 0.848, respectively, the highest among all methods, representing a considerable improvement over the second method. Its lowest AMBE of 7.45 and the best GMSD score validate the accuracy at the pixel level and in the structure.

**Table 4:** Quantitative comparison on the I-Haze dataset.

Method	PSNR↑	SSIM↑	AMBE↓	GMSD↓	VSI↑
AOD-Net (Li et al., 2017)	16.01	0.752	20.48	0.151	0.933
AMEI-Net (Zhu et al., 2021)	15.01	0.724	21.10	0.144	0.940
AMIF-Net (Galdran, 2018)	15.54	0.702	15.55	0.096	0.955
RADE (Li et al., 2021)	11.83	0.561	17.96	0.117	0.932
CEEF (Liu et al., 2022)	15.51	0.724	20.34	0.086	0.956
MSCNN (Ren et al., 2016)	15.73	0.744	19.59	0.127	0.946
AID-Net (Kulkarni and Murala, 2023)	14.54	0.670	15.70	0.175	0.936
DEA-Net (Chen et al., 2024)	13.57	0.582	15.70	0.223	0.907
PAD-Net (Yi et al., 2024)	15.97	0.745	15.77	0.154	0.929
SWAM-Net+ (Nuthi and Kankanala, 2025)	18.41	0.824	12.21	0.095	0.981
SAD-Net (Niu et al., 2025)	21.65	0.845	11.34	0.091	**0.979**
LEFTNet (Ours)	**22.28**	**0.848**	**7.45**	**0.082**	0.976

Best results in bold.

O-Haze Table 5. The most difficult one is the O-Haze benchmark, which features optically thick haze. LEFTNet achieves state-of-the-art performance with a PSNR of 24.57 dB and an SSIM of 0.846, outperforming the second-best method, SAD-Net (22.23 dB PSNR), by a notable margin. Perceptually better detail recovery is verified in extreme haze density with an AMBE of 4.92. The consistent superiority across all metrics confirms the effectiveness of the HSB for early-stage haze attenuation and of the CSFB for adaptive multi-scale feature fusion.

**Table 5:** Quantitative comparison on the O-Haze dataset.

Method	PSNR↑	SSIM↑	AMBE↓	GMSD↓	VSI↑
AOD-Net (Li et al., 2017)	16.63	0.676	20.61	0.116	0.924
AMEI-Net (Zhu et al., 2021)	14.24	0.738	49.08	0.070	**0.977**
AMIF-Net (Galdran, 2018)	16.15	0.825	34.31	0.072	0.962
RADE (Li et al., 2021)	13.18	0.491	20.02	0.209	0.827
CEEF (Liu et al., 2022)	12.22	0.490	57.25	0.144	0.901
MSCNN (Ren et al., 2016)	16.36	0.795	37.05	**0.065**	0.973
AID-Net (Kulkarni and Murala, 2023)	16.68	0.672	19.77	0.111	0.922
DEA-Net (Chen et al., 2024)	17.01	0.684	19.86	0.112	0.927
PAD-Net (Yi et al., 2024)	18.26	0.702	12.41	0.130	0.948
SWAM-Net+ (Nuthi and Kankanala, 2025)	21.73	0.834	09.10	0.069	0.975
SAD-Net (Niu et al., 2025)	22.23	0.832	8.11	0.081	0.956
LEFTNet (Ours)	**24.57**	**0.846**	**4.92**	0.067	0.976

Best results in bold.

NH-Haze Table 6. The proposed low-frequency decomposition and Channel Mining Mechanism, designed to tackle spatially varying haze, proves beneficial on this non-homogeneous haze benchmark: LEFTNet achieves the best PSNR of 21.14 dB and the lowest AMBE of 14.73. SAD-Net attains a marginally higher SSIM (0.726 vs. 0.706), indicating a small trade-off in structural similarity despite LEFTNet's overall superior haze removal and color fidelity on this challenging dataset.

**Table 6:** Quantitative comparison on the NH-HAZE dataset.

Method	PSNR↑	SSIM↑	AMBE↓	GMSD↓	VSI↑
AOD-Net (Li et al., 2017)	13.73	0.411	32.47	0.356	0.489
AMEI-Net (Zhu et al., 2021)	14.75	0.446	31.21	0.327	0.572
AMIF-Net (Galdran, 2018)	14.93	0.448	29.54	0.342	0.584
RADE (Li et al., 2021)	15.85	0.421	28.96	0.322	0.674
CEEF (Liu et al., 2022)	14.63	0.523	29.34	0.341	0.572
MSCNN (Ren et al., 2016)	15.54	0.480	27.83	0.343	0.621
AID-Net (Kulkarni and Murala, 2023)	14.63	0.441	30.22	0.256	0.599
DEA-Net (Chen et al., 2024)	17.85	0.572	21.76	0.248	0.689
PAD-Net (Yi et al., 2024)	16.25	0.512	32.48	0.325	0.682
SWAM-Net+ (Nuthi and Kankanala, 2025)	18.21	0.685	15.58	**0.191**	0.834
SAD-Net (Niu et al., 2025)	20.48	**0.726**	15.45	0.225	0.821
LEFTNet (Ours)	**21.14**	0.706	**14.73**	0.192	**0.894**

Best results are shown in bold.

### 4.3 Qualitative analysis

In addition to quantitative metrics, we also perform a visual quality analysis to evaluate perceptual performance. Color fidelity, texture preservation, haze density uniformity, and the presence of artifacts are not necessarily captured by scalar metrics as clearly as they appear in the images.

RESIDE-6K Figure 5. The hazy input shows a thick, uniform whitish veil, especially in the upper parts of the buildings and the sky. AOD-Net is successful at removing haze but results in significant color distortion (an unusually dark sky and a blue-green tint on facades). Residual haze is noticeable in RADE and PAD-Net. DEA-Net adds strong over-saturation. There is slight over-saturation in MSCNN. SWAM Net+ and SAD-Net achieve good dehazing with minor residual imperfections. AMIF-Net and AMEI-Net make reasonably faithful color renditions with a small cool spectral shift. LEFTNet can remove haze thoroughly and correctly, leaving a clear blue sky with a natural gradient, the warm brick-red colors of building facades, sharp building contours, and detailed roof contours, in the best approximation to the ground truth.

**Figure 5:**
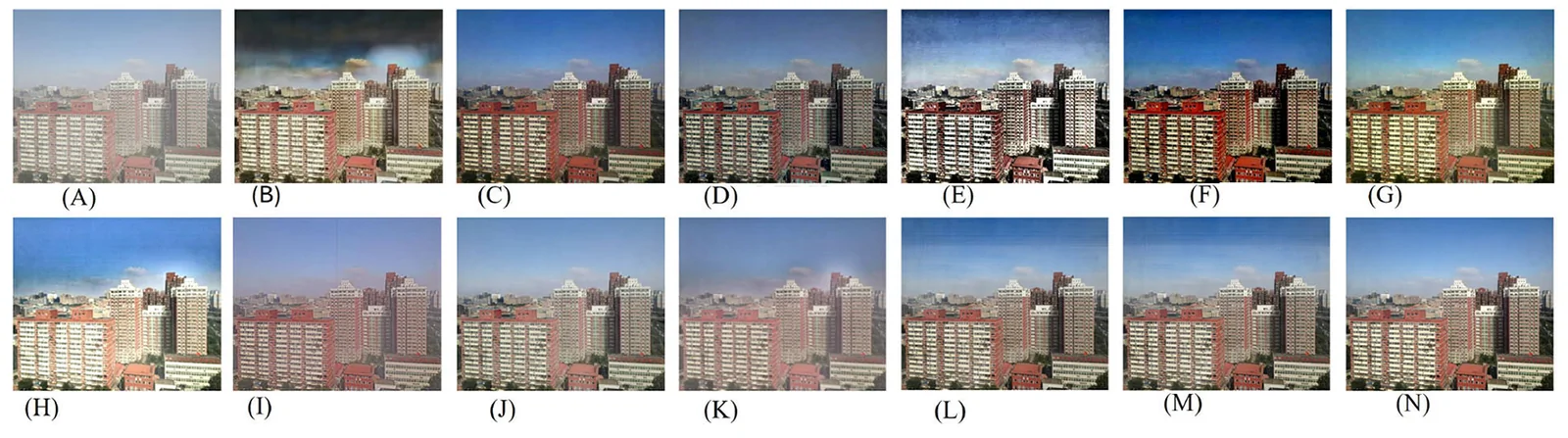
Qualitative comparison on the RESIDE-6K dataset. **(A)** Hazy. **(B)** AOD-Net. **(C)** AMEI-Net. **(D)** AMIF-Net. **(E)** RADE. **(F)** CEEF. **(G)** MSCNN. **(H)** AID-Net. **(I)** DEA-Net. **(J)** PAD-Net. **(K)** SWAM-Net+. **(L)** SAD-Net. **(M)** Ours. **(N)** GT.

I-Haze Figure 6. AOD-Net leaves visible haze residue. AMEI-Net and AMIF-Net produce color changes on the floor and wall. RADE has a blocky distortion and produces a darker output. CEEF is aggressive and brightens up the scene, washing out the background boundaries. MSCNN produces cleaner output; however, the dark door region is not well recovered. AID-Net and DEA-Net over-smooth the scene, blurring the bicycle textures and room structure. With PAD-Net, residual haze can be observed at a certain altitude above the ceiling. SWAM Net+ and SAD-Net effectively reduce haze, but slight color shifts remain. LEFTNet recovers bicycle structures, floor surface, and background door with natural clarity and accurate colors, providing the closest match to the ground truth.

**Figure 6:**
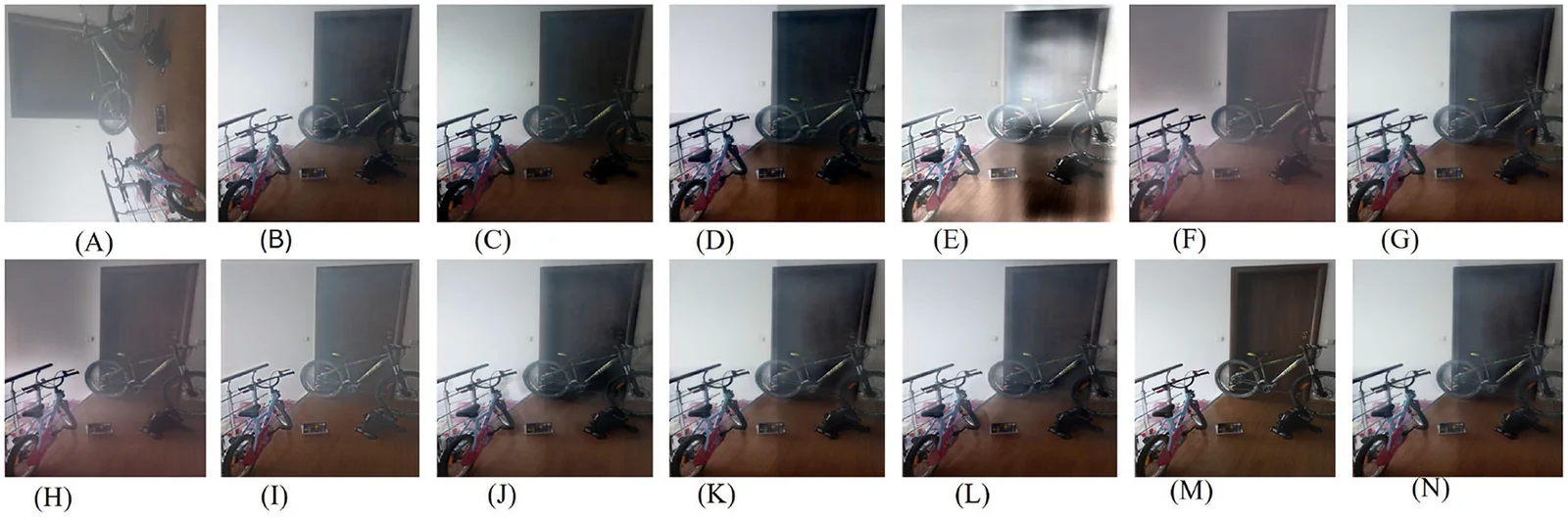
Qualitative comparison of dehazing results on the I-Haze dataset. **(A)** Hazy. **(B)** AOD-Net. **(C)** AMEI-Net. **(D)** AMIF-Net. **(E)** RADE. **(F)** CEEF. **(G)** MSCNN. **(H)** AID-Net. **(I)** DEA-Net. **(J)** PAD-Net. **(K)** SWAM-Net+. **(L)** SAD-Net. **(M)** Ours. **(N)** GT.

O-Haze Figure 7. When used against a white backdrop, AMEI-Net will darken the sky and add a muted tone to the overall image. AMIF-Net presents a cool cast for building facades, with improved near-field visibility and reduced background haze. CEEF over-enhances contrast. PAD-Net gives a minor reddish tint. DEA-Net exhibits non-uniform dehazing and color distortion. Background buildings are tightly wrapped with RADE, which performs the poorest. SWAM Net+ and SAD-Net improve scene visibility, though minor haze residues remain. LEFTNet is a method that reconstructs the scene of building surfaces, sky area, and background structures with realistic colors and clarity, closely matching the ground truth.

**Figure 7:**
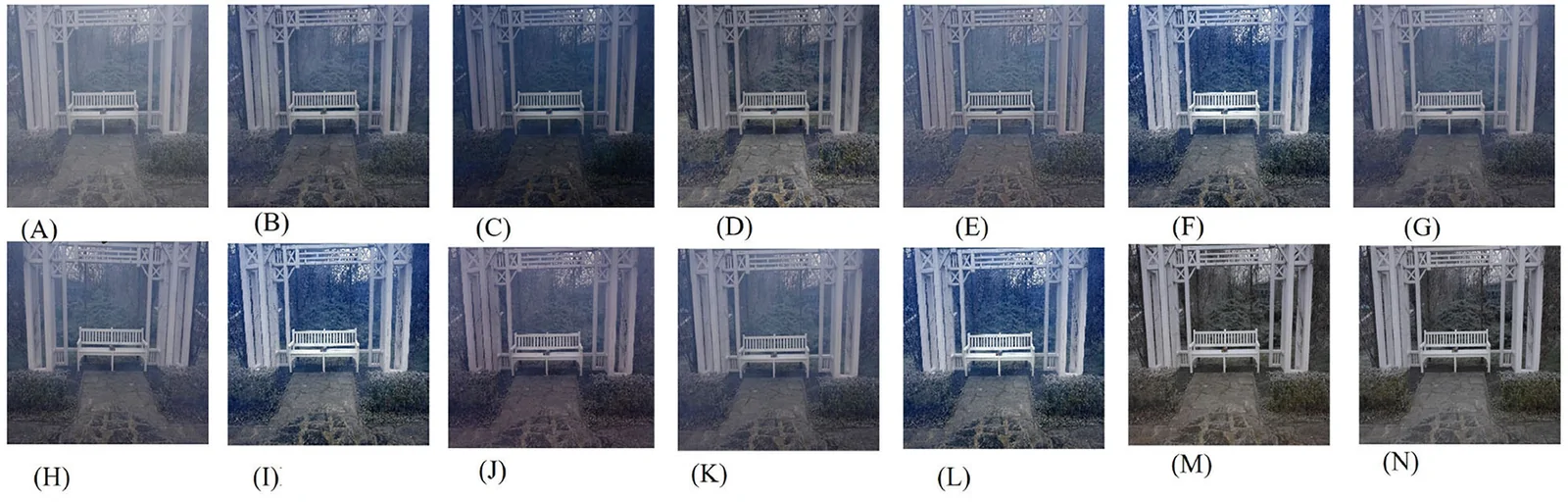
Qualitative comparison of dehazing results on the O-Haze dataset. **(A)** Hazy. **(B)** AOD-Net. **(C)** AMEI-Net. **(D)** AMIF-Net. **(E)** RADE. **(F)** CEEF. **(G)** MSCNN. **(H)** AID-Net. **(I)** DEA-Net. **(J)** PAD-Net. **(K)** SWAM-Net+. **(L)** SAD-Net. **(M)** Ours. **(N)** GT.

NH-Haze Figure 8. The test image depicts a garden with a white gazebo and bench. AOD-Net partially clears the haze, but it has a flat appearance and lacks details of the bench and pathways. AMEI-Net introduces a greenish color cast. AMIF-Net has an interesting blue-green color. The effect of RADE is to change the scene to a yellowish appearance, but it cannot handle spatially varying haze. CEEF is capable of creating a very dark and blue print. For MSCNN and AID-Net, the results are dark blue, with little detail on the bench. In the central region, DEA-Net produces an overexposed bright patch because dense haze is misidentified, resulting in the loss of background foliage detail. SWAM Net+ and SAD-Net recover details well, but subtle artifacts are visible in complex regions. LEFTNet achieves the best visual fidelity and an accurate white gazebo frame, recovering the stone pathway and bench structure, as well as the surrounding foliage with natural green hues and colors that most closely resemble the ground truth.

**Figure 8:**
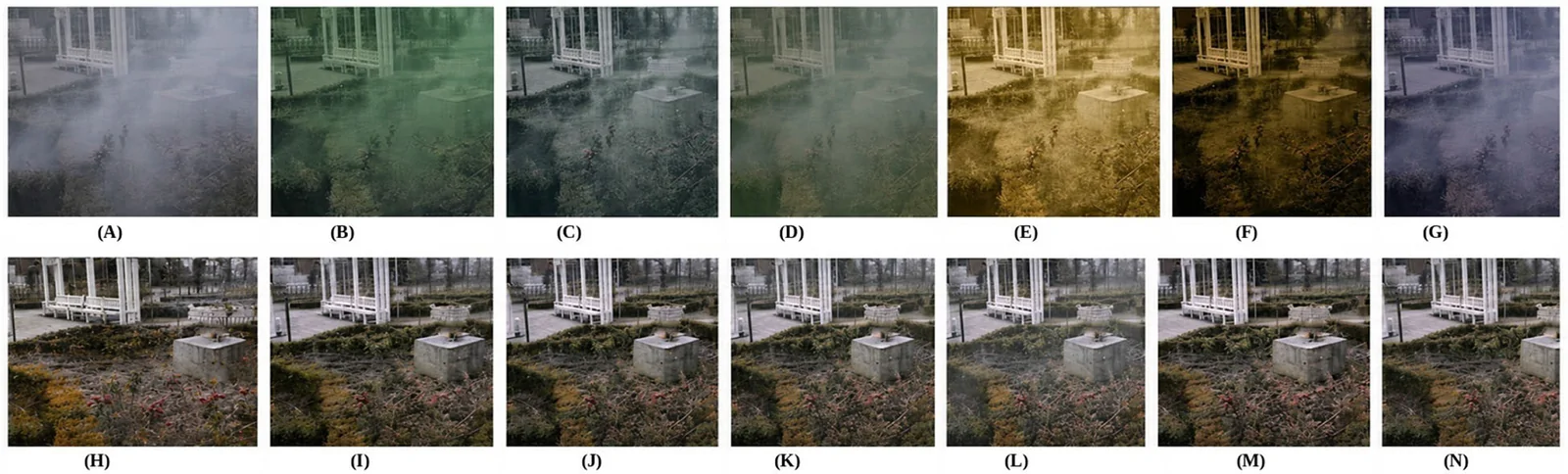
Qualitative comparison on the NH-HAZE dataset. **(A)** Hazy. **(B)** AOD-Net. **(C)** AMEI-Net. **(D)** AMIF-Net. **(E)** RADE. **(F)** CEEF. **(G)** MSCNN. **(H)** AID-Net. **(I)** DEA. **(J)** PAD. **(K)** SWAM-Net+. **(L)** SAD-Net. **(M)** Ours. **(N)** GT.

Real-Haze (Nuthi and Kankanala, 2025; Li et al., 2019) Figure 9. As ground-truth data is unavailable, a qualitative comparison is conducted using real-world aerial images of cityscapes. AOD-Net adds a nice reddish-pink hue. AMEI-Net gives an overly dark output. The scene changes to an unnatural teal with AMIF-Net. RADE is a deep blue. CEEF and MSCNN exhibit higher brightness recovery but poorer color consistency. AID-Net and DEA-Net remove the scene content and the haze. PAD-Net has relatively high visibility, with a slight warm-sky shift. SWAM Net+ and SAD-Net enhance visual quality, yet slight distortions persist under haze conditions. LEFTNet delivers the most natural-looking results, featuring sharp building contours, nice skyline tones, and no color distortion.

**Figure 9:**
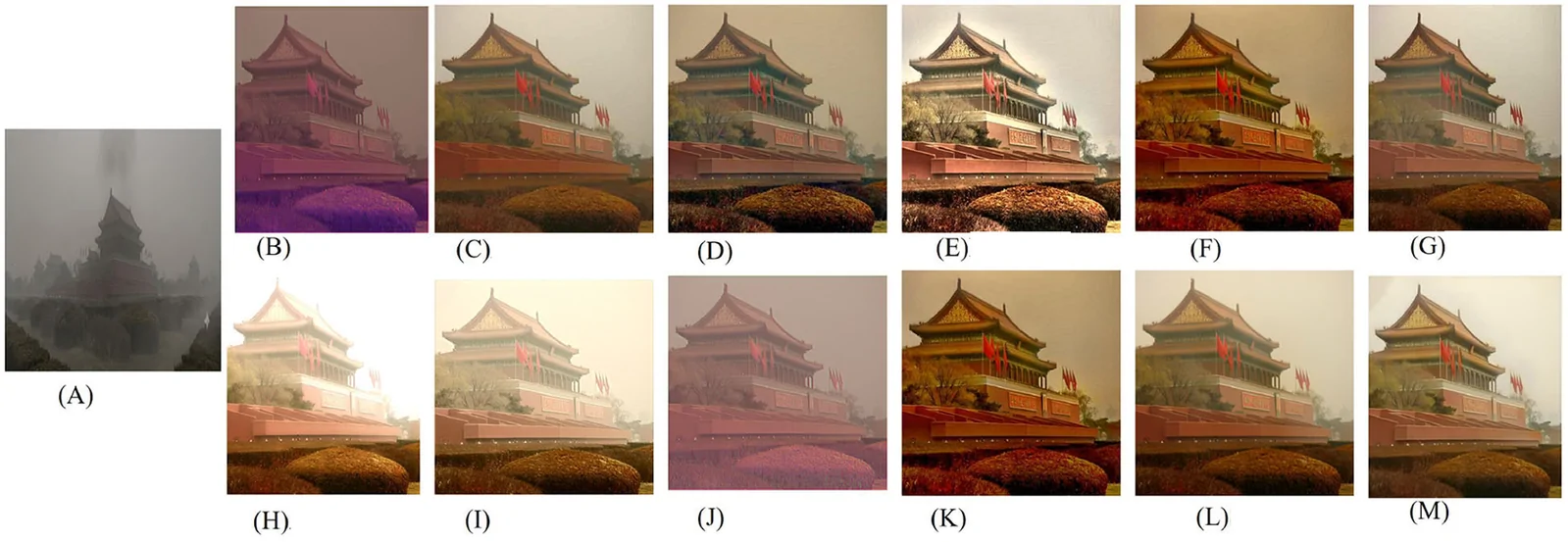
Qualitative comparison on the Real-Haze dataset. **(A)** Hazy. **(B)** AOD-Net. **(C)** AMEI-Net. **(D)** AMIF-Net. **(E)** RADE. **(F)** CEEF. **(G)** MSCNN. **(H)** AID-Net. **(I)** DEA-Net. **(J)** PAD-Net. **(K)** SWAM-Net+. **(L)** SAD-Net. **(M)** Ours.

### 4.4 Ablation study

To perform the ablation study systematically, we carry out a detailed study on NH-Haze for eight configurations: the full model and the variants obtained by removing one module from the model at a time, Table 7.

**Table 7:** Ablation study on NH-Haze.

Configuration	Params (M)	PSNR (dB)↑	SSIM↑
Full Model (LEFTNet)	2.747	21.14	0.706
Single-branch (parameter-matched)	2.747	20.27	0.693
w/o HSB	-	19.84	0.694
w/o DBB	-	19.21	0.681
w/o CSFB	-	19.56	0.688
w/o Channel Mining	-	18.73	0.667
w/o HSB + DBB	-	18.95	0.673
Baseline (Enc-Dec only)	-	17.84	0.642

Contribution of CSFB. Removing CSFB reduces the PSNR to 19.56 dB and the SSIM to 0.688. If adaptive multi-scale fusion is not applied, the decoder lacks multi-scale features to learn fine structural details and global haze context simultaneously from the shallow and deep stages, respectively. The performance gap validates the significance of adaptive integration of cross-scale features for spatially varying haze.

Contribution of Channel Mining. The PSNR decreases to 18.73 dB when the Channel Mining module is excluded. This notable performance reduction (2.41 dB) underscores the importance of channel-aware feature refinement. By adaptively emphasizing informative channels after multi-scale feature aggregation, the module improves haze-related feature discrimination and enhances restoration quality. These results demonstrate that Channel Mining plays a key role in achieving LEFTNet's overall effectiveness.

Combined ablations. The complementarity of the two modules is evident: ablating DBB yields the largest degradation (PSNR 19.21 dB, a 1.93 dB drop), indicating its importance for early input-level haze suppression. The ablation of HSB results in a PSNR of 19.84 dB, demonstrating its role in refining features from global to local representations. Furthermore, removing both HSB and DBB reduces the PSNR to 18.95 dB, a 2.19 dB drop from the full model, highlighting the complementary contributions of the two modules. As shown, the baseline encoder–decoder system, without the proposed modules, achieves only 17.84 dB PSNR, indicating that the full multi-module design is necessary to achieve the superior performance of LEFTNet. The parameter-matched single-branch model in Table 7 is used exclusively for performance ablation against the full dual-branch model. In contrast, the standard single-branch model in Table 2 is used for computational complexity comparison. These represent two distinct single-branch configurations.

### 4.5 Computational complexity and efficiency analysis

To further substantiate the computational efficiency of the proposed Dual-Branch Block (DBB), Table 1 compares its measured computational cost against a hypothetical full self-attention mechanism operating over the flattened *H*×*W* spatial grid at the three feature scales used in the network. Although the correspondence maps produced by DBB retain a term quadratic in the spatial width (*W*^2^), the block batches attention over the height dimension (*b*·*h*) rather than folding it into the attention itself, so its complexity scales as *O*(*H*·*W*^2^·*C*) rather than *O*(*H*^2^·*W*^2^·*C*) for standard full self-attention. This design choice yields an 85–341 × reduction in Multiply-Accumulate Operations (MACs) across the three scales (297 × overall), confirming that DBB avoids the full quadratic blow-up of transformer-style attention while still modeling cross-branch correspondence.

Table 2 reports the parameter count, MACs, and FLOPs of the complete dual-branch network relative to a single-branch variant with the DBB cross-branch fusion removed, at 512 × 512 input resolution. Since the encoder and decoder weights are shared across the left and right branches, the parameter overhead introduced by DBB is limited to 0.261 M parameters, while the additional computational cost arising from DBB's internal convolutional layers and its BMM-based attention operations brings the total from 182.69 to 421.60 G FLOPs. These results provide the quantitative model-complexity comparison that was previously missing from the manuscript and place the added cost of cross-branch fusion in context relative to the shared single-branch trunk. The measured runtime increases from 163.59 ms for the single-branch variant to 409.98 ms for the full dual-branch model. Runtime is averaged over 50 forward passes on an NVIDIA T4 GPU.

### 4.6 Limitations

Despite achieving state-of-the-art PSNR and AMBE across all evaluated datasets, LEFTNet does not consistently outperform all baselines on structure-similarity-oriented metrics such as SSIM and GMSD. In particular, PAD-Net achieves stronger SSIM and GMSD on RESIDE-6K, and SAD-Net achieves marginally higher SSIM on NH-HAZE, suggesting that these methods' structure-preservation mechanisms may be better suited to specific haze characteristics. We attribute this gap to the DBB module's emphasis on cross-view color/illumination correspondence rather than fine-grained structural alignment, and identify this as a direction for future work, potentially via an explicit structure-preserving loss term.

## 5 Conclusion

We introduced a single-image dehazing network, named LEFTNet, which consists of four synergistic modules: Haze Suppression Block (HSB), Dual-Branch Block (DBB), Cross-Scale Fusion Block (CSFB), and Channel Mining Mechanism (CMM). LEFTNet explicitly separates low-frequency haze components based on atmospheric scattering principles, thereby gradually and systematically eliminating haze at each level. The HSB suppresses channels with haze early; the DBB matches the channel feature scale by adaptively fusing multi-scale features via learnable coefficients; and the CMM performs second-stage channel recalibration to further suppress channels with haze. Complementary representation learning and joint accuracy and perceptually natural outputs are achieved by a symmetric dual-branch architecture and hybrid $L_{1}+L_{SSIM}$ loss. The results of comprehensive experiments on the four benchmark datasets (RESIDE-6K, NH-Haze, I-Haze, and O-Haze) and tests on real images show that it achieves competitive results compared with 11 state-of-the-art methods across the evaluation metrics. Ablation studies have verified that each module has a vital and unique function. The main difference between LEFTNet and other models is its ability to achieve brightness fidelity and preserve gradients. These two features are most directly related to the quality of photorealistic output, as shown in the comparison tables (AMBE and GMSD) for almost all datasets. Future research needs include increasing LEFTNet's capability in dense and thick haze, enhancing its depth awareness and perceptual consistency in extreme non-uniform haze situations, and developing lightweight versions for real-time use in autonomous systems.

## Data Availability

The raw data supporting the conclusions of this article will be made available by the authors, without undue reservation.
